# Instructions for Mount’g Teeth by Casting

**Published:** 1858-04

**Authors:** 


### INSTRUCTIONS FOR MOUNTING TEETH BY CASTING MORE RECENTLY DENOMINATED CHEOPLASTY.

The following is an *exact* copy of Dr. Blandy's instructions to the purchasers of rights.

Take the impression as usual in wax or plaster: plaster being preferred from its greater accuracy, as the superior fit results from the metal giving the exact counterpart of the impression.

2. Surround the impression as high as its margin, with softened putty. It should be so surrounded as to allow the placing of a *tin rim*.

1. For deep palates, raise the centre of the impression cup with wax. Some using a spatula to cover the roof of the mouth with plaster. Salt should be used in plaster to *quicken* the set. Sulphate of potash, is said to be better, being free of taste.

2. The impression if of plaster, should have the central cavity or chamber cut at this time, and varnished, to afford an easy delivery of the cast. If of wax, the chambers cut with a sharp blade, suitable for the purpose, and slightly warmed, thus any form of cavity is easily given.

Great care should be taken, to use a ring that will surround the impression fully ¼ inch, and at least 1 inch or 1½ inches behind the termination of the ridge, to allow a partition surface for the two parts of the matrices to divide upon, and to afford depth for gate, as well as for articulating surfaces needed in taking the bite.

The impression having previously been varnished, should now be oiled, as well as all exposed surfaces inside the ring, which is now prepared to receive the plaster and spar, in equal weight well mixed with water, about the thickness of cream, when poured, stir with a camel's hair *pencil*, or *feather*, into the impression until it begins to *set*, then fill to the depth of ¾ of an inch, if properly done it will come away from the impression *perfectly smooth*. When hard, remove the ring and putty, and carefully separate the cast from the impression, which need not be sacrificed. In cases of projecting alveolus, the impression may be cut through, until the color of the varnish is reached at those points, where the ridge binds, then by gently tapping the part cut, it will break so as to be readily replaced, and thus permit any number of casts to be taken from the same impression.

Dr. Slayton recommends hydraulic cement, instead of spar,—finely ground quartz sand may be used.

The air cavity can now be altered, trimmed and made perfectly smooth. This cavity should be formed previous to taking the composition cast, but if omitted can be formed by placing a flattened plate of wax with the size and form of the desired chamber cut from it, the thickness of the wax representing the depth of cavity. Press the wax down carefully upon the cast, the chamber being placed in its proper position, moisten the surface of the cast within the wax, and pour plaster and spar to fill the space. When hard, warm the wax and remove it, leaving a good substitute for a chamber formed in the impression.

Casts of the foregoing are not as hard as of *plaster only*, but sufficiently so, and may be hardened by using Fuch's soluble glass.

To form a pattern for plate and afford means for articulation, wax rolled into sheets 1-40 inch thick is used.

First protect the cast by a plate of tin-foil, such as is used by druggists, modeled down upon it with the finger assisted by a hard roll of soft leather, the sheet wax is then carefully modeled down upon the tin-foil plate and neatly trimmed to the size and extension of the plate—for upper sets form a second tin-foil plate upon the wax, then place a rim of wax for the impression of the lower teeth as in old processes, this rim must be carefully trimmed to the desired length and fullness. To stiffen the plate, warm a small ball of wax until it becomes soft then press it with care over the surface of the upper tin plate inside the rim, cool it in water forming a plate that the most difficult articulation can be taken with certainty. Taking it from the mouth, place it on the cast and form two or three small holes upon the part extending behind the plate for articulating surface. Build putty around the outside and a little above the rim of wax carrying the impression of the lower teeth, and extended back behind the cast to surround the part where holes have been made. Oil the surface within the putty and carefully fill with plaster. When hard remove the putty and carefully separate the model of lower teeth from the composition cast, cut off wax rim, remove the wax stiffener and tin upper plate together, and you have the wax pattern plate ready to receive the teeth.

It would be well to place upon the foil plate before fitting the wax one or two narrow strips of foil over the prominent ridges of the alveolus, so that in adjusting the teeth the proper space may not be decreased from under them, which would exclude the metal from flowing at such points, which is almost unavoidable where the alveolar border is sharp.

The process for obtaining the articulation in double sets is much the same as in other methods. The plates being made as described, a tin-foil plate on both casts followed by the wax plate which is again covered by foil, to protect it from injury—adhesion of the wax, and admit of easy removal, rims of wax are neatly trimmed and modeled to these plates, strengthened by strips of wax, or better as before described, that is filling inside the rim softened wax to cover the whole plate.

To strengthen the lower plate, if the wax is not sufficient, take a narrow strip of any metal, bent into a circle to fit along the inside of the plate as low as convenient, cover and unite to the plate by melted wax, the metal rim being bent before the plate is disturbed, if the outside of the plate is broad it should be stiffened by making the rim of wax to cover it. The plates are placed in the mouth, and the rims of wax altered if necessary to represent the length of teeth desired, and made to close so as their whole surfaces strike evenly at the same time.

The patient should be made to close the mouth with a *careless* rapidity, the operator can thus see the true position and mark the wax rims by lines crossing at various points, commencing with the median line. The plates are removed and placed together as in the mouth, and firmly held to this adjusted position by melted wax. Place the upper plate upon its cast with the under one attached, fill in with putty the space inside the plates back to the articulating surface, as high as the inner edge of the lower plate, then with the aid of the tin rim placed around the upper cast, extending a little above the outside edge of lower plate, then all unnecessary spaces filled with putty, the whole surface oiled or washed with thick soap water and plaster poured in, which forms with the addition of but one cast the correct articulation for a double set. Of course the usual process can be used if preferred—wholly of plaster casts, or with the hinged metal articulator; after hardening and separating, remove the rims of wax and stiffening, leaving the wax plates ready for the adjustment of the teeth.

It would be well to add a strip of soft wax to the rims used in obtaining the bite so that the rims or the lower teeth may imbed themselves freely. In fitting the teeth to the plate they require, generally, only lateral grinding which must be accurate, except when the prominences of ridge make them too long, when they may be cut down preserving the outer or gum surface perfect. As each tooth is ground to accommodate its neighbor, it should be held in its position by a small quantity of melted wax, run under its base, from the point of the shaping tool, care being observed not to use too much so as to form a lump, hold the tooth firmly with as little wax as possible. After all are fitted accurately, pass a narrow strip of wax plate around the the inside of the teeth, its width being indicated by the length of crowns from the plate, then with the small leather dub or brush-press the wax carefully between the necks of the teeth, cutting off all superfluous wax, and finishing as smoothly as possible. A narrow strip is now carried around the outside resting on the wax plate, to form a rim, covering the ends of the gum, a little melted wax holds this strip all around and the warm knife smooths it partially down, when the wax is made as smooth as possible a small blow-pipe and spirit lamp are used, and by short gentle puffs the wax is made to flow presenting a beautifully polished surface. Upon this depends all the labor of finishing, for after a little practice it becomes the work of a moment. At this stage it is best to try the teeth in the mouth to correct any-oversight or omission. When satisfied on all points place at once upon the cast, the plate carefully pressed down at every point with the brushes, close adaptation of the wax plate is absolutely necessary, if done well the plate needs no support, otherwise it must be supported in its position by tying a thread over the molars and around the cast, the tin rim by which the cast was made is now placed around projecting some distance above the teeth, the surface of the cast inside of the rim and outside of the wax plate freely oiled or painted with strong soap water to make it part freely, and the upper flask or matrix forced little by little carefully in, of the same composition as the other, covering the teeth to the depth of a quarter of an inch, excluding all air bubbles, and securing the absolute filling of the spaces around and between the teeth, by aid of a camels hair pencil or feather, with which it should be kept stirred until it begins to set, when hard remove the rim and tap the part first made—gently—until the two parts loosen when they may be separated.

The gate should always be cut from the part containing the teeth and plate, and done when removed before wax plate is taken out, to prevent small pieces falling in, etc., the wax plate and as much of the wax as possible should be removed, for although the flask will absorb it, yet the melted and absorbed wax softens and weakens the composition inducing a roughness of the plate, the metal should always be poured fast into the mould, vents cut at the highest point or last run. To obtain a smooth surface and to aid the metal in flowing, hold the surfaces over a gas light, or candle flame until they are slightly covered with carbonaceous matter from the smoke. After all the wax is removed, gate and vents cut, the two parts of the mould are placed together and firmly bound with iron wire, then carefully lute the surface of joint with plaster and spar from one vent to the other, as well as the under surface of impression cast to give it strength. The piece is now placed in a bake-oven of a stove or range or any vessel in which a good bread baking heat can be obtained, the heat should be uniform, and range from 300 to 400° F., (but never allowed to come in contact with a red hot surface or flame, this would induce cracks, warpage, or too great softness, and produce leakage of hot metal when poured), and exposed to this heat three hours, or longer if possible, to be made thoroughly dry.

The metal should be melted and made hot until of a light blue color, the cast hotter than can be held in the hand, should be placed in an upright position, the metal poured rapidly in the gate until the vent is filled, should never bubble, as this is certain evidence of moisture, the great necessity being to have your flasks *perfectly dry* and the *metal hot.* When cold, the part containing the teeth should be cut off from around them, and thus easily removed exposing the plate upon the cast, at which time any repairs can be made by the use of solders Nos. 1 and 2, and muriate of zinc as a flux, but with care it never becomes necessary.

Partial sets and plain teeth are mounted with comparatively less proportionate time than whole sets. The articulation of one or more teeth should be done in the mouth after grinding and fitting and the piece stiffened as described, when adjusted to the cast the plaster teeth should be carefully cut off and surfaces made smooth so as to allow an easy separation of the two parts.

*Repairing.*—Cut out the broken part, and file away the metal freely, fit in a tooth, and build around it wax as you desire the metal to be, make a gate by rolling a piece of wax round or oval shaped, and tapering from one-half to three-sixteenths inches, fix the small end to the largest surface of wax, which has been built around the tooth, from the opposite highest point make a vent of wax ⅛ inch, and have it so placed as to stand on a level with the gate, place a tin rim around the plate and fill in with composition carefully as before, when hard, remove as much of the wax forming the gate and vent as possible, and place the piece to dry at a *less heat than will melt the metal.* When thoroughly dry, pour the metal in and allow it to cool, and it is ready for dressing. All alterations are expeditiously done by simply adding wax to the plate as the desired alterations require, involve the whole in composition, after forming a gate and vent as described, drying and pouring.

The operator should bear in mind, that he is modeling an exact representation of the piece, and that the slightest imperfection will be faithfully copied.

Every thing will depend upon the *entire absence of moisture*, and the proper heat of the metal represented by its turning a *light blue* color. After the piece is cool, the gate and vent should be cut off, with a spring saw, and dressed off with scrapers, emery paper, or any instruments the operator may prefer, and finally polished or burnished as other pieces. Emery, fine pumice stone, or spar, used with water, and finally dry, answers best.

*Resetting old plates*, particularly for the lower jaw, which from absorption cease to be worn with comfort. Take the impression of the jaw, proceed as described in obtaining the articulation, prepare the rejected piece, by cutting off the plate as close to the teeth as possible, but allowing them to remain united with each other, then place them firmly on the wax plate, and cover the backing with wax to envelope all the old metal, place a narrow strip of wax around the outside, smooth all down with the knife and blow-pipe, having articulated correctly. With continuous gum, cut all the platina off, and place on the wax plate—mould a delicate strip all around the edge of the gum, so as slightly to imbed the set, proceed as for upper, drying, pouring, &c.

Soldering is readily done, by either of the solders prepared. No. 1, is brittle and not so pure, and flows at about one-half the melting point of the plate metal, it should be used as sparingly as possible, by fitting in pieces of plate metal in all places to be repaired, using only sufficient solder to unite the two parts. Pieces can be held in the hand whilst using No. 1, and the place to be repaired gradually heated to the melting point of the solder, by the small blow-pipe, by gentle puffs until it flows suitably. No. 2, is elastic and strong, and requires greater heat to fuse it, and greater care to prevent melting the plate.

*Teeth* should never be soldered in repairing, but always poured, as the solder will incline to run to the metal, and not take hold of the tooth.

*Blow-pipe.*—This should be very small, requiring considerable pressure to force the air through, otherwise you endanger your wax-plate in polishing, and your metal in soldering, by throwing too much heat or covering too much surface.

*Gating.*—Secure a good size gate in length and thickness, if the plate is to be very thin, let the gate extend into the plate some distance, by cutting out from its surface a small gate-groove extending half way over the chamber. Giving an increased thickness of plate at this point which can easily be cut away after pouring.

*Venting* should be well secured by presenting a free opening upon both sides of the gate, so that air and gas may pass without obstruction.

*Polishing, Wax.*—This should be done with great care, so that the wax shall not run over the teeth, or in spots where the metal is not required.

### MATERIALS AND TOOLS.

I am prepared to supply the profession with, materials and tools for the CASTING PROCESS, viz:

Oliver & Harrison's gum teeth, 20 cents each.

Plaster, spar, hydraulic cement, sand, ground soap stone, putty, wax, tin foil, &c.

ALSO.

| | |
|---|---|
| Scrapers, 6 different patterns, .......................... | 35 cents each. |
| Burnishers, .......................................... | 50 " " |
| Modeling Tools, ...................................... | 50 " " |
| Blowpipes, ........................................... | 25 " " |
| Tin rings or frames, | |

Casting metal, $1,00 per ¼ pound, $3,00 per pound Troy.

Solder 50 cents per ounce.

ALSO.

Iron Flasks, at $1,50. These however are not necessary.

